# A Combination of Near-Infrared Hyperspectral Imaging with Two-Dimensional Correlation Analysis for Monitoring the Content of Alanine in Beef

**DOI:** 10.3390/bios12111043

**Published:** 2022-11-18

**Authors:** Fujia Dong, Yongzhao Bi, Jie Hao, Sijia Liu, Yu Lv, Jiarui Cui, Songlei Wang, Yafang Han, Argenis Rodas-González

**Affiliations:** 1 School of Food and Wine, Ningxia University, Yinchuan 750021, China; 2 Department of Animal Science, Faculty of Agricultural and Food Sciences, University of Manitoba, Winnipeg, MB R3T 2N2, Canada

**Keywords:** hyperspectral imaging, two–dimensional correlation spectroscopy, alanine, visualization

## Abstract

Alanine (Ala), as the most important free amino acid, plays a significant role in food taste characteristics and human health regulation. The feasibility of using near–infrared hyperspectral imaging (NIR–HSI) combined with two–dimensional correlation spectroscopy (2D–COS) analysis to predict beef Ala content quickly and nondestructively is first proposed in this study. With Ala content as the external disturbance condition, the sequence of chemical bond changes caused by synchronous and asynchronous correlation spectrum changes in 2D–COS was analyzed, and local sensitive variables closely related to Ala content were obtained. On this basis, the simplified linear, nonlinear, and artificial neural network models developed by the weighted coefficient based on the feature wavelength extraction method were compared. The results show that with the change in Ala content in beef, the double-frequency absorption of the C-H bond of CH_2_ in the chemical bond sequence occurred prior to the third vibration of the C=O bond and the first stretching of O-H in COOH. Furthermore, the wavelength within the 1136–1478 nm spectrum range was obtained as the local study area of Ala content. The linear partial least squares regression (PLSR) model based on effective wavelengths was selected by competitive adaptive reweighted sampling (CARS) from 2D–COS analysis, and provided excellent results (R^2^_C_ of 0.8141, R^2^_P_ of 0.8458, and RPDp of 2.54). Finally, the visual distribution of Ala content in beef was produced by the optimal simplified combination model. The results show that 2D–COS combined with NIR–HSI could be used as an effective method to monitor Ala content in beef.

## 1. Introduction

Beef is a popular food in the human diet, and its rich amino acid and protein composition is close to human needs [1]. With the rapid development of social economy and the continuous improvement in life quality, meat quality plays an increasingly important role in determining the value of meat products, and more and more consumers are attracted to high-quality meat [2]. As the most critical factor affecting the quality of meat products, flavor not only affects the taste of food and the absorption of nutrients, but also determines the consumers’ purchase desire and intake intention to a certain extent [3]. Free amino acids (FAA) are the main taste substances in meat products. Their type and content have an important impact on meat quality, antioxidant activity, and nutritional value. They can also be used as precursors for the Maillard reaction and Strecker degradation reaction with reducing sugar, affecting the overall flavor of the food system [4]. Different FAAs have different taste characteristics. They have a low taste threshold, strong taste ability, and five basic taste senses: sour, sweet, bitter, salty, and umami [5]. Among them, alanine (Ala), as the most simple-flavored amino acid among FAAs, has become the main sweet amino acid in meat products due to its low hydrophobicity, and the umami of food will be directly affected by its content [6]. When Ala coexisted with taste substances such as glutamic acid and ornithine in food, it can produce a synergistic effect and provide strong umami for meat products [7]. In addition, Ala also plays a variety of important physiological roles, including improving the immune system, preventing and treating vascular diseases, and participating in growth and metabolism [8]. When too much or too little Ala is ingested, the absorption balance of human total amino acids might be affected, leading to nutritional imbalance and poor health [9]. Therefore, it is of great significance to develop a rapid, nondestructive, and noncontact quantitative method for the determination of Ala content in beef.

At present, high-performance liquid chromatography and automatic amino acid analysis are often used for the physical and chemical detection of FAA [2,3,4]. These methods have the advantage of high precision in detecting the composition and content of amino acids. However, their disadvantage is that sample pretreatment is complex, harmful, and polluting, and the integrity of the sample is damaged [10]. Therefore, the rapid detection requirements in the beef mass production process cannot be met. In previous investigations, the combination of several nondestructive rapid measurement methods and chemometric methods have been applied in the assessment of amino acid content, including visible near–infrared spectroscopy, near–infrared (NIR) spectroscopy, Fourier infrared spectroscopy, and nondestructive magnetic resonance imaging [11,12,13,14]. However, these studies mainly focused on the evaluation of research objectives concerning soybean, daqu, tea, potato, and ham [11,12,13,14,15], and detection indicators such as amino acid nitrogen [12], total amino acid [15], and total volatile basic nitrogen (TVB-N) have been emphatically discussed [16]. In addition, hyperspectral imaging (HSI) technology is more widely focused in predicting other meat-related quality attributes, especially nutritional attributes (fatty acid, protein, and intramuscular fat), technical attributes (pH and water holding capacity), sensory attributes (tenderness, color, hardness, gumminess, and chewiness), freshness attributes (thiobarbituric acid reactive substances (TBARS), total biogenic amines (TBA), and myoglobin), and microbial attributes (total viable count) of meat in different parts, types, and places of origin [17,18,19,20,21,22,23,24,25]. Notably, Cheng et al. [23,24] reported that NIR–HSI had great application potential in evaluating the content of meat quality (TBA, TBARS, and fat oxidation). Through comparison, it was found that molecules with greater contributions could be detected more easily than those with smaller contributions. The above research provides the possibility to reveal NIR–HSI prediction of Ala content in meat and meat products.

However, beef, as a complex food item, has interactions among various components (proteins, amino acids, lipids, and carbohydrates) [26]. This makes it difficult to extract the NIR spectral information of the meat, and the spectral signal presents overlapping and complexity [27]. As a result, quantitative analysis by NIR has relied heavily on the application of chemometric analysis to relate the subtle spectral changes to the variations in concentrations of certain components in the analyte [28]. In previous studies, the derivative method, weight value method, principal component analysis, and feature variable extraction were the main spectral response analysis methods for studying the spectral characteristic variables of objects [29,30]. It usually requires a lot of experiments to identify sensitive variables and build a stable model effect. This is a time-consuming and iterative process, the results of which vary with experience and the chemometric methods used. Thus, a better understanding of NIR spectra and a more accurate spectral band division is conducive to the establishment of a more robust NIR quantitative model. In recent years, two–dimensional correlation spectroscopy (2D–COS) has been applied to HSI research by some researchers to improve spectral resolution by extending one–dimensional spectral signals to the second dimension [31]. By this means, the changes in subtle spectral features are analyzed and the relationship and change order of various groups are revealed [32]. This technique has been successfully applied to spectral interpretation and spectral band allocation of the lipid oxidation of meat, the damage of myofibrils, and the spectral interpretation and distribution of protein secondary structure changes in meat [24,26]. In addition, Dong et al. [33] reported that the use of 2D–COS to select the NIR–HSI continuous sensitive interval and the establishment of a deep learning algorithm have great potential to improve the accuracy of the model. This provides a new direction for our research. As far as we know, a feasibility study using NIR–HSI combined with 2D–COS analysis to detect Ala content in beef has not been previously reported.

**Figure 1:**
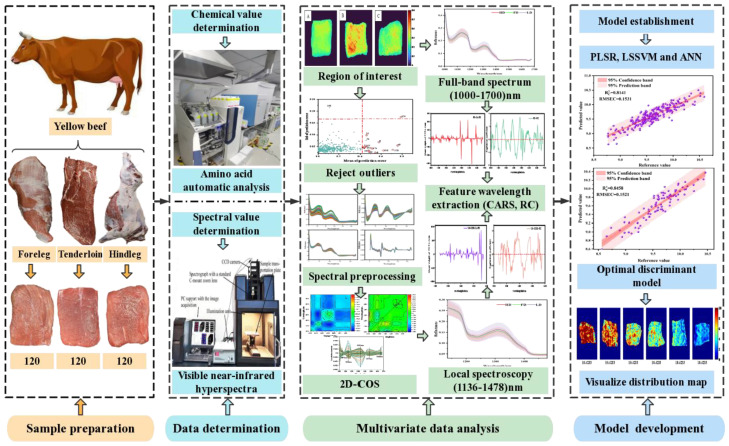
Technical roadmap of NIR–HSI detection of Ala content.

Therefore, this study is the first to explore the feasibility of NIR–HSI combined with 2D–COS analysis in detecting Ala content in beef. The specific objectives were as follows: (i) NIR–HSI (900–1700 nm) was used to collect the spectral information of beef samples, the segment threshold method was used to select the sample region of interest (ROI), and the Monte Carlo (MC) method was used to eliminate abnormal value information; (ii) the determination of the change order of characteristic peaks related to Ala content and local sensitive intervals was achieved by analyzing synchronous and asynchronous 2D–COS; (iii) the determination of the best characteristic variables of the global and local spectral intervals based on the weight algorithm (competitive adaptive reweighted sampling (CARS) and regional coefficient (RC)) were studied; (iv) simplified linear partial least squares regression (PLSR), nonlinear least squares support vector machine (LSSVM), and artificial neural network (ANN) Ala prediction models were developed; (v) the optimal characteristic variables and models obtained were used to characterize the visual distribution of Ala content. The research results were expected to further improve the accuracy of NIR–HSI technology in detecting meat quality indicators. A graphical representation of the proposed method is illustrated in Figure 1.

## 2. Materials and Methods

### 2.1. Sample Preparation

The samples were collected from three parts (longissimus dorsi (LD), foreleg (FD), and hind leg (HD)) of 20 cattle in Ningxia, China. The samples were vacuum packed and stored in a portable refrigerated incubator, and were transported to the Meat Processing and Quality Safety Control Laboratory of Ningxia University. The oil and fascia in the fresh samples were removed and cut into 40 mm × 40 mm × 20 mm (L × W × T). All samples were vacuum packed and stored in a 4 °C refrigerated room. In order to ensure the reliability and universality of the model, the samples were collected following four slaughter batches (30 samples were obtained from each part of each batch). Finally, 360 beef samples were obtained, and their spectral data and chemometric data were measured at the same time.

### 2.2. Hyperspectral Image Correction and Parameter Determination

The NIR–HSI (900–1700 nm) system was used to collect spectral images of beef samples. The HSI system can continuously acquire 256 spectral bands with a spectral resolution of 5 nm. The HSI system was mainly composed of five parts, including an HSI spectrometer, four 35 W tungsten halogen lamps, a CCD camera, an electronic displacement platform, and a computer. The best debugging parameters were optimized during preliminary experiments because diffuse reflections of the light source may be caused by the color, texture, and shape of the beef sample. The best debugging parameters were as follows: the object distance was 360 mm, the steady current of the light source was set to 6.0 A, the electric control displacement speed was 20 mm/s, and the exposure time of the camera was 30 ms. In order to reduce the uneven distribution of image light source intensity and the existence of a dark current in the sensor, black and white correction was required for the obtained hyperspectral image. The formula used was as follows:

(1) $$R=\frac{A-S}{B-S}\times 100\%\;$$

where *A* is the original spectral image of the sample; *B* is the all-white calibration image; *S* is the all-black calibration image; *R* is the calibrated spectral image. The all-black calibration image *S* was obtained by covering the camera lens (almost 0% reflectance), and the all-white calibration image *B* was obtained using a white board made of polytetrafluoroethylene (>99% reflectance). The ROI of the HSI was extracted from the spectral information of the sample using the segmented threshold method (set at 0.16) with ENVI software.

### 2.3. Measurement of the Content of FAAs

Sample pretreatment: minced meat sample (2.00 g) was weighed, 0.02 mol/L hydrochloric acid was added, and the sample was then placed in a 10 mL centrifuge tube for homogenization. After ultrasound (30 min), centrifugation (4000 r/5 min), and activation (C18), 5.00 mL methanol and 5.00 mL water were added, respectively. After filtration, 2.5 mL of solution was absorbed and 1.50 mL hydrochloric acid of 0.02 mol/L was added. After passing through the column, 0.02 mol/L hydrochloric acid was used to dilute the solution to 5.00 mL. After uniform mixing, the solution was centrifuged (10,000 r/10 min) after standing (15 min), and then filtered through a membrane of 0.45 μm pore size for analysis. By comparing the retention time and peak area of each amino acid standard, qualitative and quantitative analysis of each amino acid was carried out.

Analytical parameters: the chromatographic column used was a sulfonic acid cation resin separation column (4.6 mm × 60 mm). The detection wavelength was 440 nm and 570 nm, respectively; the injection volume was 20 μL. The reaction temperature was 135 ± 5 °C. The separation column temperature was 57 °C.

### 2.4. Analysis of Two–Dimensional Correlation Spectra

As an advanced spectral analysis method, 2D–COS is particularly suitable for exploring the structural changes and interactions of complex systems under external disturbances from the molecular perspective [31]. Compared with traditional one–dimensional spectral analysis, 2D–COS has a strong simplification effect for complex spectra containing multiple overlapping peaks. At the same time, it is extended on the basis of the original one–dimensional spectrum, significantly improving the resolution of the original spectrum [32]. With the help of a peak correlation diagram, the assignment and interaction of peaks in the system were judged, and the change order of peak position under external disturbance was obtained [26]. In this study, Ala was used as the external disturbance condition, and the dynamic spectrum $\tilde{y}$ (*v*, *d*) caused by the system in the external disturbance range (1~*T*) was defined as:

(2) $$\tilde{y}\left(v,d\right)=\{\begin{array}{c} y\left(v,d\right)-\bar{y}\left(v\right)\;1\leq d\leq T\\ \;0\;other\;wise \end{array}$$

where $\bar{y}$ (*v*) is the reference spectrum of the system, which was usually set as the average spectrum, and was defined as:

(3) $$\bar{y}\left(v\right)=\frac{1}{T}\sum_{j=1}^{T}y\left(v,d_{j}\right)\;$$

where $\bar{y}$ (*v*, *d*) data are expressed in discrete form in actual measurement. The following vector forms were commonly used:

(4) $$\bar{y}\left(v,d\right)=\left[\begin{array}{c} y=\left(v,d_{1}\right)\\ y=\left(v,d_{2}\right)\\ y=\left(v,d_{3}\right)\\ \cdot \\ \cdot \\ \cdot \\ y=\left(v,d_{m}\right) \end{array}\right]\;$$

The two–dimensional correlation intensity *X* (*v*_1_, *v*_2_) indicated the function of the spectral intensity changes $\tilde{y}$ (*v*, *d*) of the spectral variables *v*_1_ and *v*_2_, and was compared in the external disturbance variable interval. The correlation function was used to calculate the intensity change at two independent spectral variables, *v*_1_ and *v*_2_, so that *X* (*v*_1_, *v*_2_) could be converted into the plural form:

(5) $$X\left(v_{1},v_{2}\right)=\Phi \left(v_{1},v_{2}\right)\;\cdot \;i\;\Psi \left(v_{1},v_{2}\right)\;$$

According to the 2D–COS theory of Noda et al. [31,32], the mutually perpendicular real parts and imaginary parts of the complex number were called the synchronous correlation strength and asynchronous correlation strength, respectively, and the strength changes of the two were directly related to the change in d value. We then converted the dynamic spectrum from the external interference domain to the frequency domain via Hilbert–Noda change, and finally, 2D–COS was obtained. Its two–dimensional correlation synchronous spectrum was expressed as (6):

(6) $$\Phi \left(v_{1},v_{2}\right)=\frac{1}{T-1}\tilde{y}{\left(v_{1}\right)}^{T}\;\cdot \;\tilde{y}\left(v_{2}\right)\;$$

The expression of the two–dimensional correlation asynchronous spectrum was (7):

(7) $$\Psi \left(v_{1},v_{2}\right)=\frac{1}{T-1}\tilde{y}{\left(v_{1}\right)}^{T\;}\cdot \;N\;\cdot \;\tilde{y}\left(v_{2}\right)\;$$

where *N* is the *T*-order square matrix (*T* is the spectral number), which was called the Hilbert–Noda matrix; the matrix formula was (8):

(8) $$N_{jk}=\{\begin{array}{c} o_{j}=k\\ \;\frac{1}{\pi \left(k-j\right)}\;j\neq k \end{array}$$

### 2.5. Analysis Rules of Spectral Peak

The synchronous correlation spectrum characterizes the simultaneous or coincidental changes in spectral intensities measured at spectral variables of *v*_1_ and *v*_2_. In the atlas, it was symmetrical along the diagonal direction, its autocorrelation peak appeared on the diagonal, and the cross peak appeared outside the diagonal. In the synchronous spectrum, the intensity of the automatic peak was always positive, representing the overall degree of the dynamic change in spectral intensity under the corresponding number of bands. It is worth noting that there were positive and negative cross peaks in the synchronous spectrum. If the cross peaks of the two bands were positive, it meant that the spectral intensity of the corresponding wave number increased or decreased simultaneously under external interference. When the opposite value was negative, it meant that the spectral intensity corresponding to the two wave numbers increased one and decreased the other. In the asynchronous two–dimensional correlation spectrogram, the asynchronous or sequential (i.e., delayed or accelerated) changes in spectral intensity at the given wave numbers *v*_1_ and *v*_2_ were presented. Its asynchronous graph was asymmetric with respect to the diagonal, and only had cross peaks. The asynchronous cross peak only appeared when the spectral intensity of a given wave number changed out of phase. Using this feature to analyze the overlapping peaks with different sources in the spectrum had a significant role in judging the change order of the characteristic peaks in the process of external interference. The direction and order of strength change determined according to Noda rules are shown in Table 1.

### 2.6. Extraction of Spectral Characteristic Wavelength

The complex and time-consuming properties of model training were caused by the high dimension of the data and the strong correlation between adjacent variables. Therefore, the selection of characteristic wavelength variables became a key step in spectral analysis, which was mainly used to simplify the model and eliminate data redundancy. The use of genetic algorithms, principal component algorithms, iterative algorithms, and weight algorithms to extract feature variables has been reported by a large number of researchers [27,28,29]. In this study, two weight algorithms (CARS and RC) were used to select characteristic wavelengths to develop simplified models for the full spectral (FS) area and sensitive local areas selected from 2D–COS analysis. The purpose of this was to consider the proportion of data from the perspective of weight to analyze the appearance of characteristic variables more reasonably.

### 2.7. Visualization of the Ala Contents

Inversion of Ala content distribution was feasible because HSI had both image information and spectral information (combining two–dimensional imaging technology with one–dimensional spectral curve to form three–dimensional data). Based on the method of multivariate optimization model, the optimal simplified combination was selected, the weight coefficients of each pixel and the optimal correction model were calculated, and a matrix consisting of multiple predicted values was obtained. Then, the obtained matrix was refolded to generate the content distribution map [30]. The Ala content of each pixel was expressed in different color scales. Therefore, the distribution of Ala content could be clearly inverted on the color map.

### 2.8. Model Establishment and Evaluation

In this study, three multivariate algorithms, including linear PLSR, nonlinear LSSVM, and ANN, were used to develop the detection model for quantitative analysis. PLSR is a multiple factor regression method. Firstly, the scores of the main factors were extracted from the spectral matrix X and the physical and chemical matrix Y, and the PLS was used to conduct the best precision regression for the main factors of X and Y, respectively. The principal component of spectral matrix X was directly related to physical and chemical parameters, and the linear relationship between spectral variables and physical and chemical parameters was used to the greatest extent. LS-SVM used the least squares linear equation as the loss function formula. The convex quadratic programming was solved by solving linear equations instead of traditional SVM, which reduced the training time and computational complexity. ANN is a multilayer feedforward neural network characterized by forward signal propagation and backward error propagation. According to the error signal of forward propagation, the method of gradient descent was adopted for backward propagation, and the signal error was minimized through repeated forward and backward learning.

In order to establish the reliable accuracy of the validation model, the whole sample set data were divided according to a 3:1 ratio, based on the RS method. The proficiency and accuracy of the model were evaluated by analyzing statistical parameters. These included determination coefficients (R^2^), root mean square error (RMSE), and ratio of performance deviation (RPD). Generally, a good model should have higher R^2^ and RPD values and lower RMSE values.

## 3. Results and Discussion

### 3.1. Spectral Reflectance Index Visualization and Spectral Curve Analysis

**Figure 2:**
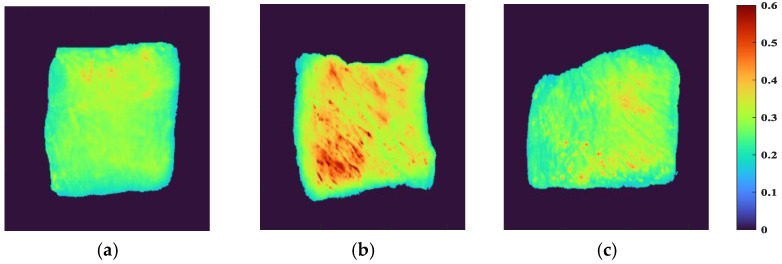
Visualization of spectral reflectance index of beef samples from different parts: (**a**) FD, (**b**) LD, and (**c**) HD.

ENVI software was used to extract sample ROI from HSI by the segmentation threshold method, and to visually express spectral reflectance indexes of different parts. First, the band with the best image definition was selected, and the optimal segmentation threshold of samples and background was set to 0.16 to retain the effective sample area as much as possible and eliminate the influence of background area interference. The independent regions of each sample were taken as ROI, and their average values were taken as the effective spectral data of the sample. The visual distribution of spectral reflectance index of beef samples from different parts is shown in Figure 2. When the color was closer to red, it meant that the reflectivity index was larger, and vice versa. It can be observed that the reflectivity index of the FD and the HD was low, and their colors were similar to each other, showing a yellow-green coloration, while the LD appeared an obvious yellow color with a small amount of red, and the reflectivity index was large. There was a certain correlation between the spectral reflectance index and the chemical composition of the sample, indicating that the LD had a more complex chemical composition than the FD and HD.

**Figure 3:**
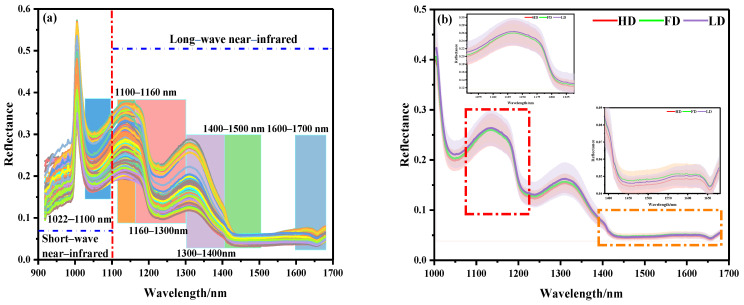
NIR spectral curves of beef samples: (**a**) full spectral curve, (**b**) spectral curves of different parts.

The spectral curves of 360 beef samples obtained by NIR–HSI are shown in Figure 3. The NIR bands of the beef samples contained rich combination bands of molecular overtones and molecular vibration, which were characterized by the double-frequency absorption of chemical bonds and relatively strong spectral characteristic absorption. As shown in Figure 3a, it was observed that there was more noise in the full-band original spectral curve of beef samples within the band range of 918–1000 nm, which might be related to the instability of the instrument at the beginning of scanning. Therefore, the spectral data of 1000–1700 nm (225 wave bands) were further analyzed. Figure 3b shows the average spectral curves of different beef samples. It can be seen that the curves show a similar trend as a whole. The spectral curve of the LD muscle was higher than that of the FD and HD, which is basically consistent with the conclusion drawn from the visualization of the ROI spectral reflectance index of the sample. It is worth noting that the longitudinal shifts of the spectra were rather different. Specifically, compared with the spectral reflectance of the 1010–1400 nm band, the reflectance of the 1400–1700 nm band was low and the peak amplitude frequency was wide. This difference might be related to the typical reflection characteristics of different components in the sample at a specific wave band when the electromagnetic radiation wave interacted with the material.

According to relevant studies, the spectral absorption band in the NIR band was mainly related to the tensile vibration of amide bonds, C-H bonds, N-H bonds, and O-H bonds of organic compounds (proteins and amino acids), lipids, carbohydrates, and water [33]. The absorption band at 1022–1100 nm might be related to the second stretching of the N-H bond in amides. The peak at 1100–1160 nm might be related to the double-frequency absorption of the C-H bond of the CH_2_ group. The absorption band at 1160–1300 nm was attributed to the second C-H stretching of carbonyl compounds. The absorption band at 1300–1400 nm represents the third vibration the of C=O bond. The absorption band at 1400–1500 nm represents the first O-H stretching of COOH. The infrared spectrum in the 1600–1700 nm region was related to the amide I band of the protein, which was mainly attributed to the absorption of the C=O bond [17,22,23,24,25,26,27,28,29,30].

### 3.2. Abnormal Sample Detection and Sample Set Division

**Figure 4:**
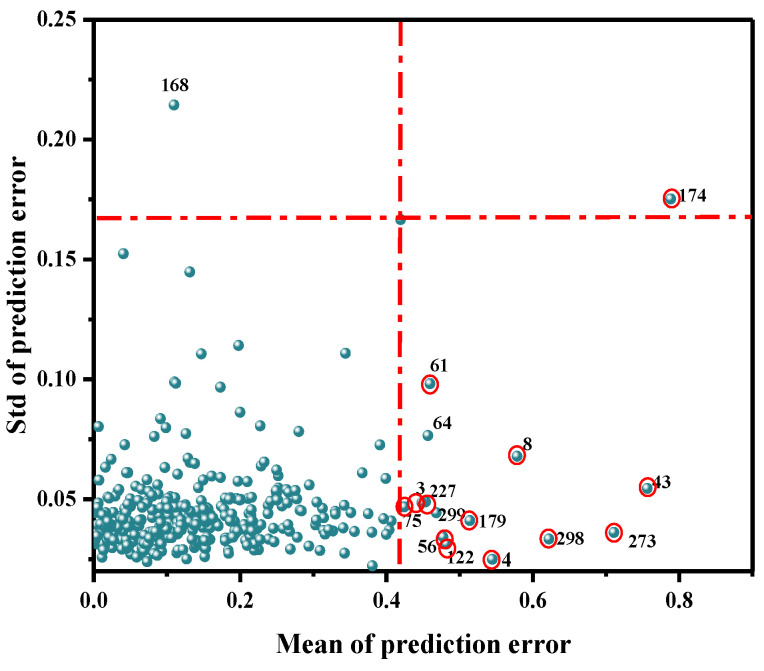
Detection results of abnormal Ala content in beef at the NIR band.

As a common method to eliminate outliers, MC can help identify the variation among samples and improve the accuracy of the model. It was mainly used to calculate the corresponding mean value and standard deviation, via a random sampling method, and to further draw the distribution map. Generally, the points far away from the main sample were assumed to be outliers, and were thus eliminated. Abnormal samples were identified based on the improvement or decrease in the performance of the established PLSR model. As shown in Figure 4, 16 abnormal value samples of 360 samples (3, 4, 8, 64, 43, 56, 61, 75, 122, 168, 174, 179, 227, 273, 298, 299) were identified by MC. The modeling effect of each abnormal value was obtained by eliminating samples one by one, as shown in Table 2. When samples 64, 168, and 299 were removed, the prediction performance of the model was reduced; therefore, false samples were retained. When the remaining 13 samples were removed, the final PLSR model was established. The R^2^_cv_ value increased from 0.6905 to 0.7419, and the RMSECV value decreased from 0.2071 mg/100 g to 0.1831 mg/100 g.

The partition of sample set was an important step in spectral multivariate analysis. After removing the outliers, 260 samples were selected as the calibration set using the RS algorithm, and 87 samples were used to predict the model performance. The statistical results are shown in Table 3. It can be seen that the range of the Ala prediction set was included in the correction set, and the difference between the average value and the standard deviation of the two datasets was not large, which means that the distribution of the divided sample sets was similar. This was highly beneficial to the establishment of a prediction model with high accuracy and robustness. At the same time, it also shows that it was feasible to use the RS method to divide samples.

### 3.3. Analysis of Spectral Full Band Modeling

In order to maximize the resolution of overlapping data and reduce the system noise caused by spectral scattering and instrument drift, nine mathematical preprocessing methods were used to correct the full spectrum data. The full wavelength data that was relevant to Ala content were evaluated and compared according to the PLSR model (Table 4). The original spectral results show a good prediction model effect (R^2^_C_ = 0.8202, RMSEC = 0.1507 mg/100 g; R^2^_P_ = 0.8145, RMSEP = 0.1663 mg/100 g). The modeling performance of the nine pretreatments had a small overall difference. Compared with the original spectrum, the PLSR model established by MF pretreatment showed the highest correlation coefficient when evaluating the Ala content, i.e., R^2^_C_ increased to 0.833, R^2^_P_ increased to 0.8388, and RMSEP decreased to 0.1548. As a nonlinear signal processing technology, MF had a good filtering effect on spectral images. At the same time, it was able to protect the edge data of the signal. Fan et al. [27] also used MF to optimize the original NIR spectrum to determine the TBA content in Tan mutton, and obtained Rc and Rp values of 0.94 and 0.88, respectively.

### 3.4. 2D–COS Analysis of Ala Content in NIR–HSI

**Figure 5:**
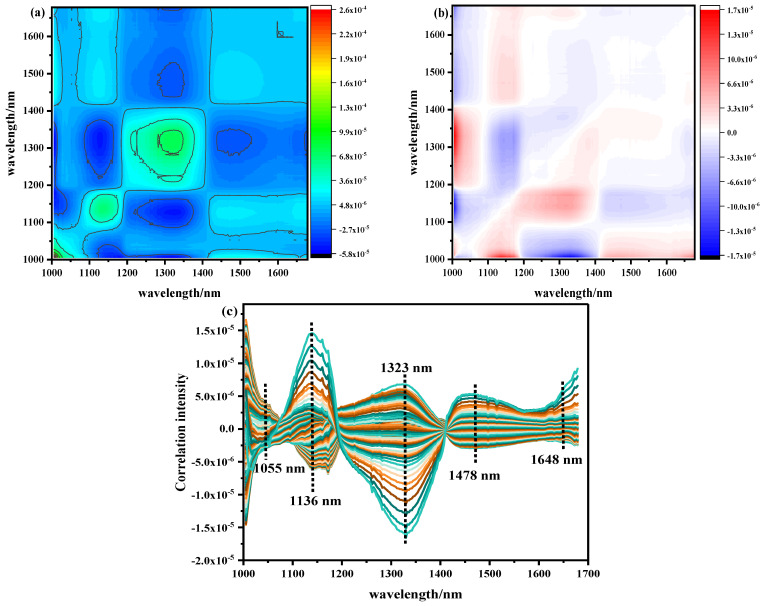
NIR two–dimensional correlation spectrum and cutting spectrum of Ala content: (**a**) synchronous two–dimensional correlation spectrum, (**b**) asynchronous two–dimensional correlation spectrum, (**c**) two–dimensional correlation cut spectrum.

The pretreated NIR with Ala content as the external disturbance condition was analyzed by 2D–COS, as shown in Figure 5a. The self-peak in the synchronous spectrum was positive on its diagonal. The strength of the automatic peak was reflected by the number of circles. The more circles, the stronger the automatic peak. On the contrary, the fewer circles present, the weaker the automatic peak. The corresponding cut spectrum in Figure 5c was generated in order to more clearly represent the position of its self-peak. It can be seen that five main self-peaks were 1055, 1136, 1323, 1478, and 1648 nm at the diagonal position. The appearance of these autocorrelation peaks indicates that with the change in Ala content, the spectral absorption intensity of the band underwent a strong change, and the spectral signals of these characteristic variables were more sensitive to external interference. Among them, 1055 nm belonged to the second stretching of the N-H bond in amides, 1136 nm was the double-frequency absorption band of the C-H bond in CH_2_, 1323 nm was the third vibration of the C=O bond, 1478 nm was the first O-H stretching of COOH, and 1648 nm was the absorption stretching of the C=O bond in the amide I band of protein [22,23,24,25,26,27,28,29,30]. Analyzing the cross peaks outside the main diagonal of the synchronous spectrum, only the obvious cross peaks were observed at (1136–1323), (1323–1478), and (1478–1136) nm. However, there was no cross peak between (1055–1136) and (1478–1648) nm, indicating that the absorption peak between them was irrelevant; thus, the self-peak at 1055 nm and 1648 nm was excluded. At the same time, it was found that the cross peaks generated by the 1136 nm and 1478 nm (named band group 1) bands were always positive, indicating that the spectral intensity of these wavelengths increased or decreased with the fluctuation of disturbance. The cross peak generated in the 1323 nm (named band group 2) band was always negative, which indicates that the bands from group 1 and group 2 demonstrated opposite behaviors.

The asynchronous 2D–COS is shown in Figure 5b. When the phase of the spectral intensity change in two variables was different, cross peaks were generated and used to infer the order of spectral intensity change. The same main cross peak was observed at the self-peak position of the synchronous spectrum. The signs of the cross peaks are shown in Table 5. According to the positive and negative signs of synchronous asynchronous cross peaks, it can be concluded that under the condition of Ala content as an external disturbance, the order of changes in the relevant spectra was 1136–1323–1478 nm. This shows that under the change in Ala content in beef, the double-frequency absorption of the C-H bond (1136 nm) in CH_2_ occurred before the third vibration of the C=O bond (1323 nm) and the first O-H stretching of COOH (1478 nm).

From the above analysis, we could see that more sensitive variables could be obtained by 2D–COS compared with the one–dimensional spectral curve. Therefore, in the further study, the sensitive range 1136–1478 nm (115 wave bands) of 2D–COS analysis was selected as the detection area of beef Ala content. Its local region contained the main absorption band of the one–dimensional spectrum. The spectral resolution was significantly improved by this method, and the spectral band contributions from different sources and the order of chemical bond changes were distinguished.

### 3.5. Characteristic Wavelength Extraction

In order to improve the prediction performance of the model to a certain extent, the feature variable screening method based on the weight algorithm was selected to evaluate the global data and local regional data of Ala. The purpose of this was to verify the impact of different weighting methods on the model effect [34].

**Figure 6:**
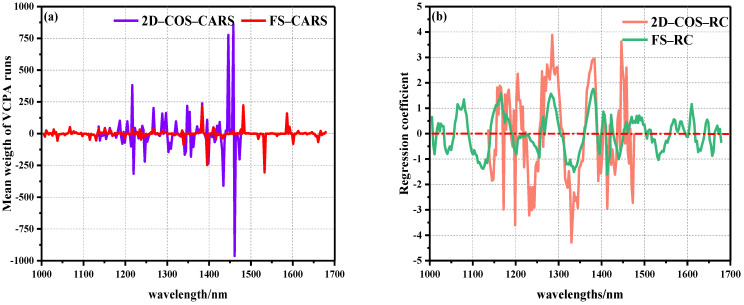
Operation diagram of characteristic wavelength extracted by weight algorithm: (**a**) average weight distribution diagram of CARS algorithm, (**b**) RC algorithm weight regression coefficient distribution diagram.

As a fast variable selection method, CARS algorithm was proposed according to the principle of “survival of the fittest” in Darwin’s theory of evolution. Due to the randomness of the MC sampling method in the CARS algorithm, in order to ensure the reliability of the model, each set MC sampling number was ran 500 times, respectively, and a 10-fold cross-validation method was used to take the wavelength corresponding to the minimum RMSECV value in all PLSR models as the optimal variable [35]. Figure 6a shows the average weight distribution of CARS. It can be seen that the characteristic variables of the whole band selected by CARS were evenly distributed, and the weight value changed slightly. On the contrary, the position of characteristic variables appearing in the local band selected by 2D–COS was denser and closer to the position selected in the full band, but the weight value was significantly higher. This shows that the local features selected by 2D–COS contained a large number of features, and the correlation between features was large. In the case of local data, the disappearance of some features would not affect the detection and matching of other features [35].

There were 36 characteristic variables selected by FS–CARS, including 1029, 1038, 1068, 1115, 1130, 1136, 1151, 1163, 1199, 1217, 1235, 1238, 1246, 1267, 1288, 1336, 1339, 1378, 1381, 1384, 1395, 1410–1416, 1431, 1437, 1446, 1458, 1476, 1482, 1518, 1533, 1586, 1592, 1601, and 1661 nm. In 2D–COS–CARS, 36 characteristic wavelengths were selected, including 1136, 1139, 1187, 1193, 1199, 1205, 1214, 1220, 1240, 1246, 1267, 1288, 1297, 1303, 1309, 1321, 1342–1348, 1354, 1357, 1363, 1372, 1384, 1395, 1398, 1413–1419, 1425, 1431, 1434, 1446, 1458, 1461, and 1473 nm. We found that when feature variables were extracted by the CARS algorithm, the number of extracted variables remained stable without significant changes in the total number of bands of the model. Wan et al. [25] also reported that in the selection of characteristic wavelengths for HSI detection of OxyMb and MetMb content in Tan mutton, the CARS algorithm was used to extract 36 and 33 characteristic variables, respectively, to establish linear and nonlinear models that provided the best results. In addition, Cheng et al. and Zhuang et al. [36,37] reported that 22 and 26 characteristic variables were extracted by the CARS algorithm to obtain effective prediction results in evaluating meat DeoMb and TVB-N content. The above reports strongly demonstrate the robustness and effectiveness of the CARS algorithm, and the number of selected feature wavelengths in these studies was between 20–40, which is similar to the number of feature wavelength extractions in this study.

The RC algorithm, as a common spectral peak analysis method, is widely used in HSI nondestructive testing. The RC algorithm conducted PLSR modeling and analysis on the reflectance vector X corresponding to each wavelength in the spectral matrix of the calibration set and the component value vector Y to be measured in the physicochemical matrix, and the wavelength regression coefficient distribution map was obtained. The larger the absolute value of the regression coefficient corresponding to the wavelength point, the more information obtained, and the stronger the correlation. Therefore, the wavelength with large absolute value of regression coefficient was selected to participate in the model establishment process [34]. Figure 6b shows the distribution of weight regression coefficients for the prediction of beef Ala content in two spectral intervals using the RC algorithm. It was also found that the local weight of 2D–COS selection was significantly higher than the global weight, and there were relatively many characteristic variables in the local area with strong significant positive correlation peaks.

In FS–RC, 26 characteristic variables were selected, of which 1005, 1020, 1080, 1165, 1285, 1381, 1404, 1422, 1488, 1568, 1601, 1610, 1646, and 1670 nm were positively correlated with Ala, and 1011, 1041, 1124, 1255, 1336, 1395, 1413, 1440, 1533, 1625, 1658, and 1679 nm showed negative correlations with Ala. At the same time, 22 characteristic variables were selected in 2D–COS–RC, among which 1165, 1184, 1193, 1195, 1205, 1264, 1285, 1381, 1407, 1425, 1446, and 1458 nm were positively correlated with Ala, and 1148, 1172, 1199, 1220, 1232, 1330, 1392, 1413, 1431, and 1473 nm showed negative correlations with Ala. In the overall range, a strong positive correlation was observed at 1079, 1165, 1205, 1285, 1381, and 1446 nm, of which the peak at 1079 nm was related to the second stretching of the N-H bond in amides. The peaks at 1165, 1204, and 1285 nm were related to the second C-H stretching of carbonyl compounds. The peak at 1383 nm represents the third vibration of the C=O bond. The peak at 1446 nm represents the first O-H stretching of COOH [22,23,24,25,26,27,28,29,30]. It is worth noting that the regression trends of the weights of the two groups of data were similar, with 1165, 1285, and 1381 nm peaks overlapping.

### 3.6. Comparison of PLSR, ANN, and LS-SVM Model Effects

In order to evaluate the effect of multicombination data model, PLSR, ANN, and LS-SVM models established by feature variable data were extracted based on the weight algorithm of FS and 2D–COS, and the results are shown in Table 6. First of all, the linear PLSR model had the best effect in FS modeling, whereby R^2^_C_ = 0.8330 and R^2^_P_ = 0.8388, which is 9.42 and 2.74% higher than LS-SVM and ANN in the prediction set, respectively. In contrast, the modeling results of 2D–COS local data in the band with 48.9% reduction were similar to those of full band modeling, and the best modeling effect was also shown in the linear model (R^2^_C_ = 0.8203, R^2^_P_ = 0.8190).

In the data modeling of feature wavelength extraction, the number of feature variables selected by RC was less than for CARS, but the modeling result was poor in the entire dataset. This was partly due to the negative correlation between the selected bands and Ala content. In addition, some useful information related to Ala content was lost in the process of manually selecting the absolute value of the regression coefficient to maximize, which made the model less adaptable. In variable selection modeling using the CARS algorithm, the same number of 36 characteristic variables were selected in FS and 2D–COS data, respectively, which effectively reduced data redundancy and obtained better model performance. Surprisingly, all models of CARS in the two data intervals were better than the original data and RC feature extraction data. This shows that the key bands determined by the CARS algorithm are rich in information and highly correlated with Ala content. The main reason for this is that CARS establishes a calibration model for each variable separately during operation, and calculates the weight of the regression coefficient in each variable. Different from RC algorithm, in order to avoid subjective selection, an exponential decreasing function (EDF) was used to reduce the variable space. Furthermore, adaptive reweighted sampling (ARS) was used to reduce the number of variables. Finally, the optimal variable quantum set with the minimum RMSECV was retained [35]. To put it another way, when the weighting methods were inconsistent, there were huge differences in the model effects.

Further discussing the modeling performance of CARS with the two datasets, we found that the three models based on 2D–COS local data were better than models based on FS data. The 2D–COS–CARS–PLSR model was the best (R^2^_C_ = 0.8141, R^2^_P_ = 0.8458). This provides the possibility that although the sensitive range of 2D–COS data compared with FS data had been reduced, the modeling effect was similar and the model accuracy had not been further improved. It was mainly used to select local intervals where data overlap and redundancy still existed. When CARS was used to eliminate useless variables, local data based on 2D–COS was effectively improved. Similarly, Fan et al. [27] used NIR–HSI to predict the TBA content of mutton, and the best prediction effect was obtained through the characteristic variable of 2D–COS local sensitive interval selected by the CARS algorithm, but the effect of 2D–COS data was slightly worse than that of FS data.

We also used RPD as the evaluation index of model performance: when RPD < 1.5, the model was invalid; when 1.5 ≤ RPD ≤ 2.0, the model could distinguish low and high content samples; when 2 < RPD < 2.5, the model could be used as a semi-quantitative evaluation sample; when RPD ≥ 2.5, the model could be used for quantitative evaluation. We found that the RPDc range of Ala overall modeling was 1.90–2.58, and the RPDp range was 1.97–2.54, which indicates that the three models had good adaptability. In particular, the RPDp of linear PLSR in 2D–COS–CARS data reached 2.54, which could effectively quantitatively evaluate the content of Ala.

### 3.7. Visualization of Alanine Content in Beef

**Figure 7:**
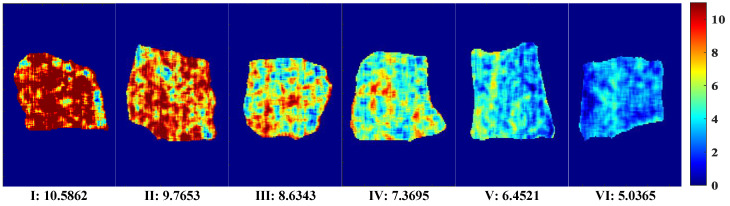
Visual distribution of Ala content.

A visual map of Ala content was developed based on the dot product of the calculated pixel points and the weight coefficient of the optimal correction model. The optimal pretreatment MF and the optimal simplified model PLSR–2D–COS–CARS were obtained through analysis. A distribution map of the predicted Ala content of each pixel was generated by multiplying the corresponding characteristic wavelength variable and the weight coefficient. The visual variation range of Ala values (5.04–10.59 mg/100 g) is shown in Figure 7. The level of content was indicated by linear chromaticity band. The red represents high value, and blue represents low value. The Ala content in the visualization chart had obvious color difference. From Figure 7(I)–(VI), the Ala content transitions from red to yellow and finally to blue, accompanied by a decrease in content. This phenomenon might be attributed to the complex changes in compounds, including protein decomposition, lipid oxidation, and water loss. In addition, some vertical stripes appeared in the Ala visualization diagram, which might be caused by jitter noise in the line scanning of the spectrum [30]. It can be observed that the visual distribution map was evenly distributed and had clear texture, and could collect more effective spectral information. Therefore, the visualization of Ala content is of great significance for intuitively and comprehensively evaluating the dynamic changes in beef quality and nutrition.

## 4. Conclusions

Ala content could be predicted by global and local data of NIR–HSI in this study. In order to improve the spectral resolution, 2D–COS was introduced to analyze the changes in spectral characteristics, reveal the change order among various groups, and find the best local research area. The spectral data of 1000–1700 nm (225 wave bands) were used as global data, and 1136–1478 nm (115 wave bands) were used as local sensitive area data of Ala content selected by 2D–COS. On this basis, in order to improve the prediction accuracy of spectral data, the adaptive effects of the two weight algorithms in PLSR, LS-SVM, and ANN models were compared. Based on the 2D–COS–CARS–PLSR model, the optimal modeling effect was achieved (R^2^_C_ was 0.8141, R^2^_P_ was 0.8458, RPDp was 2.54), which was 0.83 and 3.71% higher than FS and 2D–COS data in the prediction set. The best combination prediction model was used to generate the distribution map of Ala content prediction value. The results of this study provide a possible method to predict the Ala content of beef and better explain the changes in spectral characteristics in meat product quality.

## Figures and Tables

**Table 1:** The direction and sequence of intensity changes according to Noda’s rule.

*Φ* (*v*_1_, *v*_2_)	*Ψ* (*v*_1_, *v*_2_)	Significance
+	/	The signal strength at *v*_1_ and *v*_2_ changes in the same direction, i.e., increases or decreases at the same time.
−	/	The signal strength at *v*_1_ and *v*_2_ changes in opposite directions.
+	+	The change at *v*_1_ is mainly prior to the change in band at *v*_2_.
+	−	The change at *v*_1_ mainly follows the change in wave band at *v*_2_.
−	+	The change at *v*_1_ mainly follows the change in wave band at *v*_2_.
−	−	The change at *v*_1_ is mainly prior to the change in band at *v*_2_.

Note: *Φ* (*v*_1_, *v*_2_), synchronous correlation spectrum; *Ψ* (*v*_1_, *v*_2_), asynchronous correlation spectrum.

**Table 2:** Detection results of abnormal beef samples based on the Monte Carlo method.

Sample Set	Outliers	Remaining Amount	PB		PA	
			R^2^_CV_	RMSECV	R^2^_CV_	RMSECV
Ala	64, 168, 299	357	0.6905	0.2071	0.6209	0.2275
	3, 4, 8, 43, 56, 61, 75, 122, 174, 179, 227, 273, 298	347	0.6905	0.2071	0.7419	0.1831

Note: PB, performance before removing the outliers; PA, performance after removing the outliers.

**Table 3:** Statistical results of beef sample set divided by the RS method.

Sample Set	Calibration Set					Prediction Set				
	N	Range	Mean	SD	TAV	N	Range	Mean	SD	TAV
Ala	260	5.04–10.6	9.616	0.356	0.160	87	5.56–10.5	9.611	0.387	0.160

Note: SD, standard deviation; TAV, taste activity value.

**Table 4:** PLSR model performance of different pretreatment methods.

Sample Set	Pretreatment Method	LVs	Calibration Set		Cross-Validation		Prediction Set	
			R^2^_C_	RMSEC	R^2^_CV_	RMSECV	R^2^_P_	RMSEP
Ala	None	15	0.8202	0.1507	0.7527	0.1773	0.8145	0.1663
	MA	15	0.8156	0.1526	0.7570	0.1756	0.8230	0.1850
	GF	15	0.8180	0.1516	0.7561	0.1760	0.8287	0.1674
	**MF**	**16**	**0.8330**	**0.1452**	**0.7619**	**0.1738**	**0.8388**	**0.1548**
	SG	15	0.8129	0.1537	0.7530	0.1771	0.8191	0.1811
	Normalize	14	0.8051	0.1569	0.7510	0.1777	0.7898	0.1764
	Baseline	18	0.8136	0.1534	0.6935	0.1991	0.8165	0.1651
	SNV	13	0.7729	0.1693	0.7059	0.1934	0.7705	0.1862
	DT	17	0.8052	0.1568	0.6904	0.1997	0.8084	0.1687
	MSC	12	0.7597	0.1742	0.6941	0.1941	0.7476	0.1939

Note: Bold indicates optimal model effect. MA, moving average; GF = Gaussian filter; MF, median filter; SG, Savitzky–Golay; SNV, standard normal variate; DT, detrending; MSC, multiplicative scatter correction.

**Table 5:** Cross peak sign of two–dimensional correlation spectrum of Ala content in the NIR band.

Wavelength/nm	1136	1323	1478
Assignment	C-H	C=O	O-H
Synchronous			
1136	+	−	+
1323		+	−
1478			+
Asynchronous			
1136	\	−	+
1323		\	−
1478			\

**Table 6:** Prediction model of Ala content established by different characteristic wavelength extraction methods.

Model	Extraction Method	Variable Number	LVs	Calibration Set (n = 260)			Prediction Set (n = 87)		
				R^2^_C_	RMSEC	RPD_C_	R^2^_P_	RMSEP	RPD_P_
PLSR	FS	225	16	0.8330	0.1452	2.45	0.8388	0.1548	2.50
	FS–RC	26	15	0.7754	0.1684	2.11	0.8006	0.1721	2.25
	FS–CARS	36	18	0.8404	0.1419	2.51	0.8409	0.1538	2.52
	2D–COS	115	12	0.8203	0.1506	2.36	0.8190	0.1655	2.34
	2D–COS–RC	22	12	0.7536	0.1764	2.02	0.7919	0.1755	2.21
	2D–COS–CARS	36	13	0.8141	0.1531	2.33	0.8458	0.1521	2.54
LS-SVM	FS	225	-	0.7226	0.1876	1.90	0.7598	0.1907	2.03
	FS–RC	26	-	0.7323	0.1839	1.94	0.7446	0.1960	1.97
	FS–CARS	36	-	0.7278	0.1858	1.92	0.7596	0.1900	2.04
	2D–COS	115	-	0.8145	0.1600	2.23	0.7898	0.1657	2.34
	2D–COS–RC	22	-	0.7938	0.1704	2.09	0.7702	0.1747	2.22
	2D–COS–CARS	36	-	0.8212	0.1629	2.19	0.7980	0.1633	2.37
ANN	FS	225	-	0.8317	0.1461	2.44	0.8158	0.1650	2.35
	FS–RC	26	-	0.8275	0.1476	2.41	0.7785	0.1810	2.14
	FS–CARS	36	-	0.8492	0.1381	2.58	0.8312	0.1580	2.45
	2D–COS	115	-	0.8095	0.1566	2.27	0.7924	0.1767	2.19
	2D–COS–RC	22	-	0.7786	0.1672	2.13	0.7652	0.1863	2.08
	2D–COS–CARS	36	-	0.8484	0.1383	2.57	0.8341	0.1560	2.48

## Data Availability

The data presented in this study are available on request from the corresponding author.
